# Characterization of *GmNPF* genes prioritizes candidate genes for soybean yield and protein improvement

**DOI:** 10.3389/fpls.2026.1926264

**Published:** 2026-09-03

**Authors:** Xiaoting Wen, Le Wang, Yuan Zhang, Shoudong Wang, Dagang Wang, Xingjian Xu, Lichun Huang, Yongguo Xue, Ekaterina Shor, Yanjie Li, Hengyou Zhang

**Affiliations:** 1 Key Laboratory of Soybean Molecular Design Breeding, Northeast Institute of Geography and Agroecology, Chinese Academy of Sciences, Harbin, China; 2 University of Chinese Academy of Sciences, Beijing, China; 3 Crop Institute of Anhui Academy of Agricultural Sciences, Bio-Breeding Laboratory of Anhui Province, Hefei, China; 4 Inner Mongolia Innovation Center of Biological Breeding Technology, Hinngan League Academy of Agricultural and Animal Husbandry Sciences, Ulan Hot City, China; 5 Soybean Research Institute, Heilongjiang Academy of Agricultural Sciences, Harbin, China; 6 Institute of Plant Sciences, Agricultural Research Organization (ARO)—Volcani Institute, Rishon LeZion, Israel; 7 Heihe Branch of Heilongjiang Academy of Agricultural Sciences, Heihe, China

**Keywords:** genetic variation, GmNPFs, nitrate uptake and transport, protein content, soybean

## Abstract

Nitrate uptake and translocation are crucial for plant growth and development, with Nitrate Transporter 1/Peptide Transporter family (NRTs) being critical in mediating nitrogen supply. Soybean (*Glycine max*) has been a major source of plant-based protein globally, and it requires high amounts of nitrogen to support the plant’s development and protein accumulation in seeds. However, genes accounting for nitrogen uptake and usage have yet to be systematically identified. This study focuses on the characterization of *Nitrate Transporter 1/Peptide Transporter family* (*NPF*) genes belonging to the NRTs in soybean. In total, 126 *GmNPF* genes were identified in the soybean genome, and they exhibit evolutionary conservation and functional diversity based on the analysis of protein physicochemical properties, chromosomal localization, phylogenetic relationship, synteny correlation, conserved domain, motif, gene structure and cis-acting element prediction. Additionally, spatiotemporal expression specificity analyses revealed that several *GmNPFs* are potentially important in root, stem, leaf and seed. In addition, we also identified a set of *GmNPF* genes inducible by low nitrogen stress or being regulated by *POWR1* and *SWEET10a/b* (*SWEET39*), two major high protein QTL genes in soybean, suggesting their participation in nitrogen absorption and seed protein deposition in soybean. Importantly, we also identified six *GmNPFs* showing contrasting allelic frequencies between cultivated and wild soybean, and the allelic variation was tightly associated with the variation in protein levels in cultivated soybean. The study uncovers a set of candidate genes with great potential in improving soybean protein and yield via enhanced nitrogen uptake and utilization.

## 1 Introduction

Nitrogen (N) is an essential macronutrient that is pivotal in plant growth and development (Wang et al., 2012a). Primarily, it serves as a fundamental constituent of biomolecules, like polypeptides, amino acids, proteins, nucleic acids and chlorophyll. Its availability directly influences physiological activities in plants such as photosynthesis, protein synthesis, biomass accumulation and overall plant development (Zhang et al., 2024). In the modern agricultural system, nitrogen availability in soil is a primary limiting factor for crop yield, as increasing crop yield has been an important breeding goal to meet growing food demands worldwide (Tilman et al., 2011). However, overuse of nitrogen fertilizer would cause serious environmental concerns, enhancing the capability of nitrogen uptake, transport and use efficiency (NUE) critical in breeding practices (Ali et al., 2025). This process orchestrates a wide range of physiological and molecular processes, including sensing external nitrate availability in roots, nitrogen uptake, transport and phloem loading, particularly during the grain filling stage, which requires extensive transcriptional reprogramming enabling plants to adapt to soil nitrogen conditions or achieve successful reproductive transitions (Masclaux-Daubresse et al., 2010). Understanding the underlying molecular mechanisms is of paramount importance for enhancing nitrogen uptake and use efficiency and boosting crop productivity for achieving sustainable agricultural development.

In plants, nitrogen is primarily absorbed by plant roots in the form of nitrate ions (NO_3_^-^), which are transported into plant cells through specialized nitrate transporter proteins (O'Brien et al., 2016; Bellegarde et al., 2017). These transporters are primarily classified into the *NRT1/NPF* family (Nitrate Transporter 1/Peptide Transporter Family) and *NRT2* family (High-Affinity Nitrate Transporters) (Krapp et al., 2014; Fan et al., 2017; Li et al., 2017). The family has been investigated comprehensively in a broad range of plant species, ranging from model organisms to major crops. For example, the *NPF* family was comprehensively characterized initially in *Arabidopsis thaliana*, the genome comprises 53 *NPF* and 7 *NRT2* members (Léran et al., 2014), followed by *Oryza sativa*, including 93 *NPFs* (Kumar et al., 2022). The two studies promote the systematic analyses of *NRT/NPF* family members in numerous crops, such as 78 *NPF*, 7 *NRT2* and 2 *NRT3* genes in *Zea mays* (Jia et al., 2023), *54 NPF* genes in *Cucumis sativus* (Jia et al., 2023), 81 *StNRT* genes in *Solanum tuberosum* L (He et al., 2025), 77 *NPFs*, 7 *NRT2s* and 1 *NAR2* in *Solanum lycopersicum* (Ledesma-Valladolid et al., 2025) and 120 *GsNRT1* (*GsNPF*) and 5 *GsNRT2* genes in wild soybean (*Glycine soja*) (You et al., 2020). Other plant species with sequenced genomes were also documented, such as apple (*Malus domestica*) (Wang et al., 2018). These studies elucidate the evolution and diversification of *NRT/NPF* family, which has greatly benefited the subsequent functional validation aimed at improving nitrogen-associated traits in target crops (Léran et al., 2014).

The *NPF* family exhibits remarkable functional diversity, with nitrate absorption, transport and allocation serving as the core function in plants (Morales de Los Ríos et al., 2026). In *Arabidopsis thaliana*, AtNPF6.3/NRT1.1 exhibits bidirectional (influx/efflux) transport activity. It plays a central role in nitrogen uptake and also participates in long-distance transport of nitrate from roots to shoot (Wen and Kaiser, 2018). AtNPF4.6 and AtNPF2.7 are localized in the epidermis and cortex of root hairs, where AtNPF4.6 mediates nitrate uptake in roots under relatively high external nitrate concentrations, while AtNPF2.7 is primarily responsible for nitrate efflux from root tissues, particularly under acidic conditions (Chen et al., 2020b; Ho et al., 2009; Huang et al., 1999; Segonzac et al., 2007). In comparison, AtNRT2.4 and AtNRT2.5 function as high-affinity transporters critical for nitrate acquisition under nitrogen starvation conditions for phloem loading (Kiba et al., 2012; Lezhneva et al., 2015). The function of nitrogen uptake is conserved for many NRT/NPF across different plant species. For example, OsNPF6.5/OsNRT1.1B, OsNPF2.4 and OsNPF4.5 all serve as nitrate transporters involved in nitrate acquisition and long-distance transport within plants (Hu et al., 2015; Xia et al., 2015; Wang et al., 2020a). Similar results were also found in sweet cherry (*Prunus avium* L.), where *PaNPF5.5* overexpression enhances nitrogen uptake (Wang et al., 2023). In cucumber (*Cucumis sativus L*.), CsNPF6.4 and CsNPF2.8 are implicated in root nitrate uptake and transport to embryos (Zhang et al., 2023a). These results indicate that *NRT/NPF* genes are critical for nitrate acquisition in plant species. In addition to benefiting nitrate acquisition, *NRT/NPF* also shows environmental stress response and adaptation. For example, *SgNRT2.25* from the halophyte *Suaeda glauca* was upregulated under salt stress (Ou et al., 2025). Phosphorylation of AtNRT1.1 at Thr101 enhances drought tolerance (Kou et al., 2025). Natural variation in the promoter region of *AtNRT1.1* confers nitrogen deficiency (Sakuraba et al., 2021). Beyond its well-characterized role in nitrogen transport, the NPF family participates in mediating the transport of various phytohormones, including auxin (IAA), abscisic acid (ABA) and gibberellins (GA), as well as secondary metabolites such as glucosinolates (Chiba et al., 2015). This functional versatility underscores the *NPF* family is pivotal importance in coordinating multiple aspects of plant physiology, from nutrient allocation to stress adaptation and developmental regulation.

Soybean (*Glycine max*) is thus far the most cultivated oilseed crop worldwide that contains up to 40% protein content in seeds, making it critically important to sustain the growing demand for plant-based protein globally. Soybean growth and development require substantially greater nitrogen demands compared to other plants (Salvagiotti et al., 2008). Unlike most crops, soybeans primarily acquire nitrogen through symbiotic nitrogen fixation in root nodules. 50-60% of the nitrogen requirement in soybean is met by biologically fixed nitrogen from this symbiotic system, with the remainder obtained from soil sources (Salvagiotti et al., 2008), which could be an explanation of why the *GmNPF* gene family remains poorly characterized. Most soils are nitrogen-deficient and cannot sufficiently support optimal plant growth and development. Only a fraction, approximately as low as 30-50%, of the nitrogen fertilizer supplied into soil can be absorbed by plants (Jia et al., 2019). While supplemental nitrogen fertilization can significantly enhance soybean yield (Salvagiotti et al., 2009), excessive nitrogen fertilizer application leads to environmental pollution. Based on previous studies as mentioned above, enhancing the intrinsic capacity of plants to acquire, transport and utilize nitrogen from the soil would be a solution to mitigate the pressure from ecosystem pollution due to excessive application of nitrogen fertilizer in soils. *NPF* genes may represent the most promising candidate to boost a plant’s ability to acquire more nitrogen from the soil. Considering the ability of nitrogen fixation and less nitrogen fertilizer application during soybean growth, *NPF* genes have not received adequate attention in the soybean research community. Instead, recent studies have demonstrated that enhancing soybean nitrogen supply by optimizing nodulation or enhancing nitrogen transport efficiency could achieve a simultaneous increase of both yield and protein content (Chen et al., 2019; Liu et al., 2020b), suggesting that optimizing nitrogen uptake or utilization represents an alternative way of fertilizer application to boost yield. However, the *NPF* family in the soybean genome has not been comprehensively characterized, and few of which were further investigated.

In this study, we retrieved all putative *NPF* genes in the soybean genome and comprehensively characterized the characteristics, including chromosomal distribution, gene structure, domain organization, cis-acting elements in promoters and evolutionary relationships. We investigated the expression patterns of the family, including the expression in different tissues, with close attention to those specifically expressed in developing seed coats and seed compartments, and further identified low N stress *GmNPFs*. We also identified *GmNPFs* that were differentially expressed in soybean varieties with high and low protein levels, as well as transgenic soybean plants of *POWR1* and *SWEET10a/b* (*SWEET39*), two major QTL genes controlling high protein content in soybean (Goettel et al., 2022; Zhang et al., 2020). This study reveals a set of underexplored *GmNPFs* that might be involved in enhancing NUE or increasing protein content in soybean, which lays a molecular foundation to boost soybean yield through NUE improvement.

## 2 Materials and methods

### 2.1 Identification and physicochemical properties of the GmNPF gene family

The reference genome sequence and GFF3 annotation file (*Glycine max Wm82.a2.v1*) of soybean were downloaded from EnsemblPlants (http://plants.ensembl.org/info/data/ftp/index.html). The Hidden Markov Model (HMM) profiles of PTR2 (PF00854) were downloaded from the Pfam database (http://pfam.xfam.org/), which represented NPF. Using *Arabidopsis* NPF proteins as query sequences, the BLAST GUI Wrapper in TBtools version 2.012 (Chen et al., 2020a) was used to search transmembrane amino acid transporter domains against the protein sequences of the soybean genome (E-value was set to 1e-35 to determine the significant hits). The resulting putative NPF proteins were further confirmed manually by the NCBI Conserved Domain Database (https://www.ncbi.nlm.nih.gov/Structure/bwrpsb/bwrpsb.cgi) (Lu et al., 2020) and manually confirmed with SMART (https://smart.embl.de/). The physicochemical properties of GmNPF proteins were analyzed by Protein Parameter Calc of TBtools.

### 2.2 Phylogenetic tree construction, chromosome localization and collinearity analysis

Clustal W version 2.1 was used to compare the full-length amino acid sequences of NPFs in *Glycine max* and *Arabidopsis* with default parameters (Zhao et al., 2012; Okumoto et al., 2002). MEGA 11 was used to analyze phylogeny using the neighbor-joining (NJ) method with the following parameters: *Jones-Taylor-Thorn* model, pairwise deletion and 1000 bootstrap replications, with the result being modified by iTOL (https://itol.embl.de/) (Tamura et al., 2021). Information about gene location and tandem replication of the *GmNPF* gene family was illustrated using the program Gene Location Visualize from the GTF/GFF of Tbtools. Collinearity analysis was performed using MCScanX with an *E*-value set to 1e-5 (Wang et al., 2012b). The result of collinearity was shown by the program Advanced Circos in TBtools. The ratio of synonymous mutation to non-synonymous mutation (Ka/Ks) between gene pairs was calculated by Simple Ka/Ks Calculator of TBtools. Subcellular localization of GmNPF proteins was predicted using WoLF PSORT (https://wolfpsort.hgc.jp/) with default parameters (Horton et al., 2007).

### 2.3 Analysis of conserved motif, domain and gene structure of the GmNPF proteins

Conserved motifs of the GmNPF proteins were analyzed through MEME (http://meme.nbcr.net/meme/cgi-bin/meme.cgi) with the default parameters, in which the site distribution was patterned on zoops and the number of motifs was set to fifteen (Bailey et al., 2009). Domain analysis was performed by NCBI Batch CD-Search (https://www.ncbi.nlm.nih.gov/Structure/bwrpsb/bwrpsb.cgi) with default parameters. Data on gene structure were retrieved from the downloaded GFF3 file, and all results were visualized with Gene Structure View (Advanced) in TBtools.

### 2.4 Promoter analysis

The upstream 2000 bp base sequences of the soybean *GmNPF* genes were extracted using the program GXF Sequence Extract of TBtools. PlantCARE (https://bioinformatics.psb.ugent.be/webtools/plantcare/html/) was applied to predict cis-regulatory elements (Lescot et al., 2002). The resulting data were visualized with Basic Biosequence View in TBtools.

### 2.5 Tissue-specific gene expression

The tissue-specific and developmental expression profiles of soybean NPF genes were retrieved from the SeedGeneNetwork database (http://seedgenenetwork.net), which integrates publicly available RNA-seq datasets of seed development from multiple plant species. The expression data of all identified *GmNPF* genes across various tissues (roots, stems, leaf, floral bud, globular stage, heart stage, cotyledon stage, early maturation stage, mid maturation stage, late maturation stage, dry seed, globular stage endosperm, heart stage endosperm, cotyledon stage endosperm) were obtained by querying the database with gene IDs. The expression values (in FPKM/TPM) were log_2_-transformed and subjected to hierarchical clustering based on Euclidean distance. Heatmaps were generated using the pheatmap package in R (v4.4.1) to visualize the expression patterns (Kolde, 2019).

### 2.6 Plant materials and treatment

Soybean seeds (variety Dongnong 50, DN50) were germinated in vermiculite, following which, seedling roots were washed with tap water, and cotyledons were removed before seedlings were transferred to plastic boxes containing a foam board with 40 holes for hydroponics. The hydroponic solution containing 0.25 mM potassium dihydrogen phosphate (KH_2_PO_4_), 2.5 mM calcium nitrate (Ca(NO_3_)_2_), 2.5 mM potassium nitrate (KNO_3_), 1 mM magnesium sulfate heptahydrate (MgSO_4_·7H_2_O), 0.082 mM iron ethylenediaminetetraacetic acid (Fe-EDTA), 0.02313 mM boric acid (H_3_BO_3_), 0.00038 mM zinc sulfate heptahydrate (ZnSO_4_·7H_2_O), 0.00157 mM copper sulfate pentahydrate (CuSO_4_·5H_2_O), 0.00457 mM manganese chloride tetrahydrate (MnCl_2_·4H_2_O), and 0.000567 mM sodium molybdate monohydrate (Na_2_MoO_4_·H_2_O) was used as the normal nitrogen (NN) condition to represent controls (Hoagland and Arnon, 1938; Winter and Rostás, 2010). The nitrogen content of the low nitrogen hydroponic solution (10% of that of the normal nitrogen hydroponic solution) was used as a low nitrogen (LN) treatment, in which the missing Ca^2+^ and K^+^ were replaced by CaCl_2_ and K_2_SO_4,_ respectively. Once the plants reached seedling stages with the first fully developed triple compound leaf, the seedlings cultured in NN solution were transferred to LN solution for LN treatment (Hao et al., 2011). At 12 and 24 h post-treatment, the roots were harvested individually and were flash frozen in liquid nitrogen and stored at -80°C until RNA isolation. The roots from two or three plants were pooled as one replicate, and the experiment was set up for three biological repetitions.

The *POWR1* overexpression (*POWR1*-OE) and *GmSWEET39* knockout (*SWEET39*-KO) mutants were generated in the Williams 82 (W82) genetic background (Goettel et al., 2022; Wang et al., 2020b; Sun et al., 2025). For the POWR1-OE construct, the full-length coding sequence of *POWR1* was inserted into the pTF101.1 binary vector under the control of the CaMV 35S promoter (Paz et al., 2004). The *GmSWEET39*-KO mutant was created using the CRISPR/Cas9 gene editing technology as previously described (Sun et al., 2025). Both transgenic lines were obtained via *Agrobacterium*-mediated soybean cotyledonary node transformation following a standard protocol (Paz et al., 2006). Putative transgenic T_0_ plants were initially screened using QuickStix Strips for PAT/bar (EnviroLogix, USA) with fresh leaf tissue, and positive plants were transferred to pots and grown to maturity in the greenhouse. To identify homozygous lines, T_1_ seeds were sown in the greenhouse, and genomic DNA was extracted from the third trifoliate leaf using the CTAB method (Doyle, 1987). For *POWR1*-OE and *SWEET39*-KO lines, positive transgenic plants were initially identified by PCR-based genotyping through amplification of the BAR gene sequence. For the *SWEET39*-KO mutant, primers flanking the target site were designed, and Sanger sequencing was performed to confirm the presence of targeted mutations and to determine homozygous or heterozygous. This screening process was continued through successive generations until homozygous, stable T_4_ mutant lines were obtained. For transcriptome analysis, seed coats were collected at 30 days after flowering (DAF) from all mutant lines. For both *POWR1*-OE and *SWEET39*-KO lines, three independent transformation events were included for each genotype. For each independent transformation event, seed coats from three individual plants were pooled as one biological replicate. All samples were immediately frozen in liquid nitrogen and stored at −80 °C until RNA extraction.

Four soybean cultivars with distinct genotypes and protein contents were planted in Harbin, Heilongjiang Province, China. These cultivars included Kennong 34 (KN34) and Wodou5hao (WD5), containing low protein content, and two varieties, Suinong 120 (SN120) and Suinong 88 (SN88), containing high protein content in seeds. All cultivars were grown under standard field management conditions. At 30 days after flowering (DAF), seed coats were collected from each cultivar. Three independent biological replicates were collected per cultivar, with each replicate consisting of pooled tissues from three individual plants. The collected samples were immediately frozen in liquid nitrogen and stored at −80 °C for subsequent RNA extraction.

### 2.7 RNA extraction, RNA Sequencing, and quantitative real-time PCR

Total RNA was extracted from all samples described above, including root tissues under different nitrogen conditions, seed coats of *POWR1*-OE and its wild-type control, seed coats of *GmSWEET39*-KO and its wild-type control, and seed coats of KN34, WD5, SN120, and SN88, using an ultrapure RNA kit (CWBIO, China) following the manufacturer**’**s instructions. The NanoDrop 2000 (Thermo Scientific, USA) and 1% agarose gel were used to examine the quality of extracted RNA.

For transcriptome sequencing, a portion of the total RNA was sent to Majorbio Bio-pharm Technology Co., Ltd. (Shanghai, China) for library construction and sequencing. Briefly, mRNA was enriched from total RNA using oligo(dT) magnetic beads through A-T base pairing with poly-A tails, followed by fragmentation into approximately 300-bp fragments using fragmentation buffer. First-strand cDNA was synthesized with random hexamer primers using reverse transcriptase, and the resulting double-stranded cDNA was end-repaired and A-tailed at the 3’ ends. After adapter ligation, the fragments were size-selected and amplified by PCR to generate the final cDNA libraries. The libraries were quantified using the QuantiFluor^®^ dsDNA System, pooled at appropriate ratios, and subjected to bridge PCR amplification on a cBot system to generate clusters, followed by paired-end 2 × 150 bp sequencing on the Illumina platform. Raw sequencing reads were quality-controlled using fastp software (v0.23.2) to remove adapter sequences, low-quality reads (Phred quality score < 20), and reads containing ambiguous bases (N), resulting in clean data with Q20 > 85% and Q30 > 80%. The clean reads were then aligned to the soybean reference genome Williams 82 assembly 6 version 1 (Wm82.a6.v1) using HISAT2 (v2.2.1) with default parameters. Gene expression levels were quantified as fragments per kilobase of transcript per million mapped reads (FPKM) using featureCounts (v2.0.1). Differential expression analysis was performed using the edgeR package (v3.36.0), and genes with |log_2_(fold change)| ≥ 1 and false discovery rate (FDR) < 0.05 were identified as differentially expressed genes (DEGs).

The remaining RNA was reverse-transcribed into cDNA using a reverse transcription kit (TransGen Biotech, China) and subsequently used for quantitative reverse transcription polymerase chain reaction (qRT-PCR) validation of the selected differentially expressed NPF genes. Primers were designed by Primer Premier 5, and their specificity was checked by NCBI Primer-BLAST (https://www.ncbi.nlm.nih.gov/tools/primer-blast/index.cgi?LINK_LOC=BlastHome). qRT-PCR reactions were performed on LightCycler**^®^** 480 (Roche, Switzerland). The reaction volume was 20.0 μL, including 10.0 μL of 2×*PerfectStart****^®^*** Green qPCR SuperMix, 0.4 μL of each primer (10 μM), 1.0 μL template (10× diluted cDNA liquid), and 8.2 μL nuclease-free water. The thermal cycling program was: 94 °C for 30 s, and 40 cycles of 94 °C for 5 s; and 60 °C for 30 s. All qRT-PCR analyses were performed with three independent biological replicates, and each biological replicate contained three technical replicates. Expression of the genes was calculated using the 2^-ΔΔCT^ method (Livak and Schmittgen, 2001), and the soybean *TUB4* gene (GenBank accession no. NM_001252709) was used as the internal reference gene. All gene-specific primers were listed in the **Supplementary Materials**.

### 2.8 Genetic diversity analyses and phenotype comparison

To elucidate the domestication patterns of *NPF* family genes in soybean, we conducted comprehensive genetic diversity analyses by comparing wild soybean (*Glycine soja*) and cultivated soybean (*Glycine max*) (Zhang et al., 2022). We retrieved the coding sequences (CDS) of all *NPF* genes from both *G. soja* and *G. max* accessions and identified allelic variations between the two species. Genes exhibiting allelic differences exceeding 75% between wild and cultivated soybeans were selected for subsequent haplotype analysis. Haplotype analysis was performed using the “geneHapR” package in R (Zhang et al., 2023b). For each candidate gene, haplotypes were constructed based on the identified polymorphic sites within the CDS region. To assess the functional consequences of haplotype variation, we compared phenotypic differences among distinct haplotypes in *G. max* accessions, focusing on three key agronomic traits: seed oil content, seed protein content and 100-seed weight. Statistical comparisons were conducted using one-way ANOVA followed by Tukey’s HSD test (*P* < 0.05) to determine significant differences among haplotype groups. These analyses enabled the identification of potentially favorable alleles that may have been selected during soybean domestication and improvement.

## 3 Results

### 3.1 Genome-wide identification of GmNPF family genes in glycine max

To identify all *NPFs* members in soybean, the Hidden Markov Model (HMM) of PTR2 (PF00854) was used to screen the annotated protein database from the *Glycine max* reference genome (Wm82.a2.v1). After initial identification followed by manual confirmation, a total of 126 GmNPF proteins were identified (**Supplementary Table 1**). We named the *GmNPF* members according to their closest gene models in *Arabidopsis*. The subfamily exhibits dramatic variation in the protein length, ranging from 115 to 637, which led to a broad molecular weight span from 40.06 kDa to 71.12 kDa (**Supplementary Table 1**), implying that the family might experience extensive evolutionary divergence and functional diversification. The isoelectric point (pI) of GmNPFs ranged from 5.19 to 10.04, with 86.5% of the GmNPF proteins being alkaline (pI>7) and the remainder being acidic. In addition, we identified that nearly all of the GmNPF proteins were hydrophobic (GRAVY > 0), characteristic of membrane proteins, whereas GmNPF5.4 and GmNPF7.9 were hydrophilic (GRAVY < 0), implying functional specialization in aqueous environments (Kyte and Doolittle, 1981). Investigation of the instability index for the family reveals that GmNPF8.7, with the value of 22.28, was the smallest among all GmNPF proteins, while GmNPF6.6 ranked the highest in instability index at 60.53. Within the GmNPFs, 23 GmNPF proteins were identified as unstable proteins with instability indices exceeding 40 (**Supplementary Table 1**), indicating their potential susceptibility to degradation under physiological conditions, whereas the remainder maintained stable conformations as evidenced by their lower instability scores. Subcellular localization prediction revealed that 93% of GmNPF proteins localize to the plasma membrane, where they mediate substrate exchange between the cell and the external environment. A small subset of GmNPF proteins were predicted to target chloroplasts, the endoplasmic reticulum, cytoplasm, and the extracellular space, indicative of functional diversification within the family (**Supplementary Table 1**). Collectively, these results demonstrate that the GmNPF family members display remarkable divergence in their physicochemical properties, which may lay a solid foundation for their functional differentiation and diverse physiological roles during soybean growth and development.

### 3.2 Chromosomal localization, phylogeny and collinearity analysis of GmNPF genes

**Figure 1:**
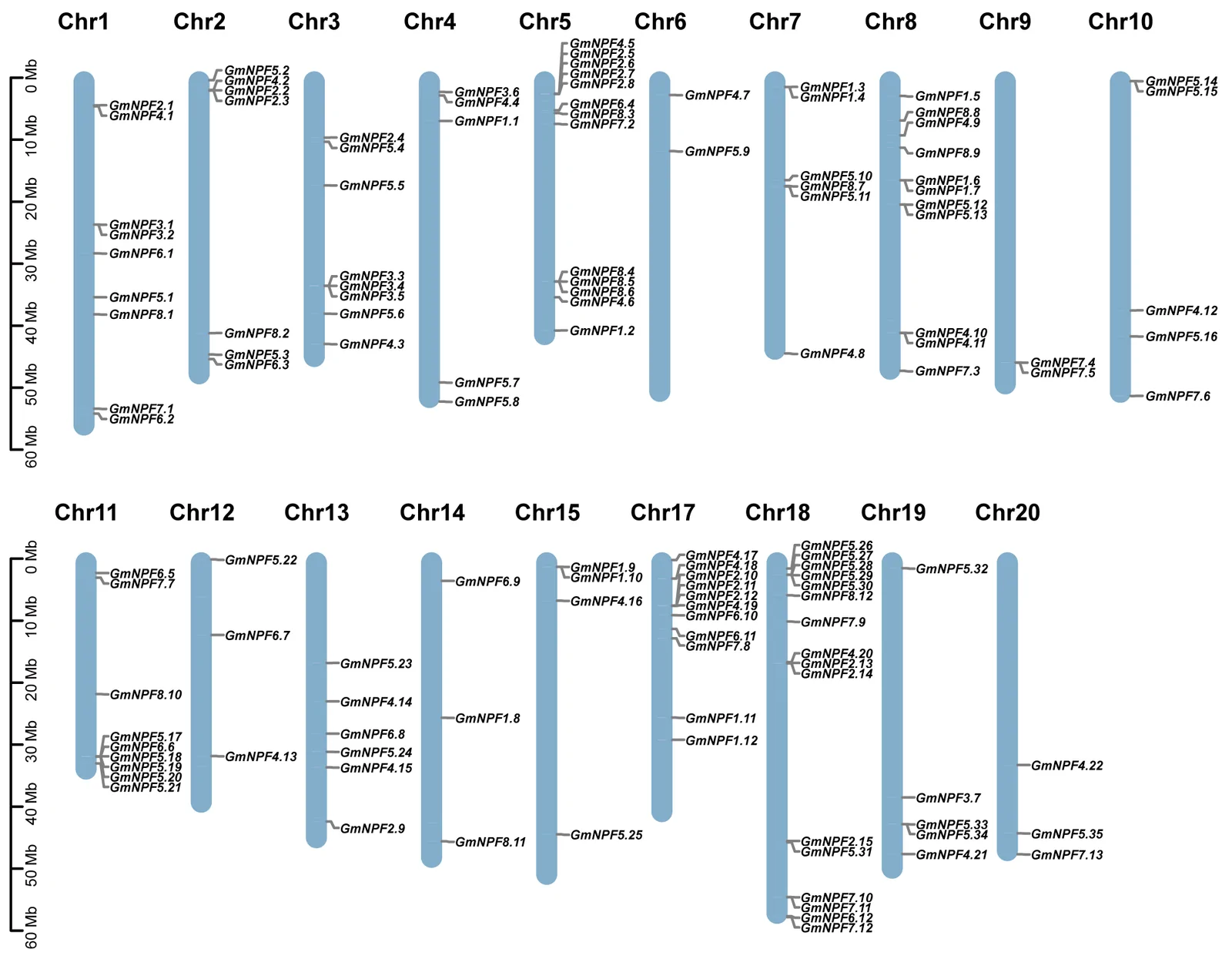
Chromosomal localization map of *NPF* genes in soybean (*Glycine max*). Chromosome numbers are labeled on the top of each chromosome, with the vertical scale in megabases (Mb).

**Figure 2:**
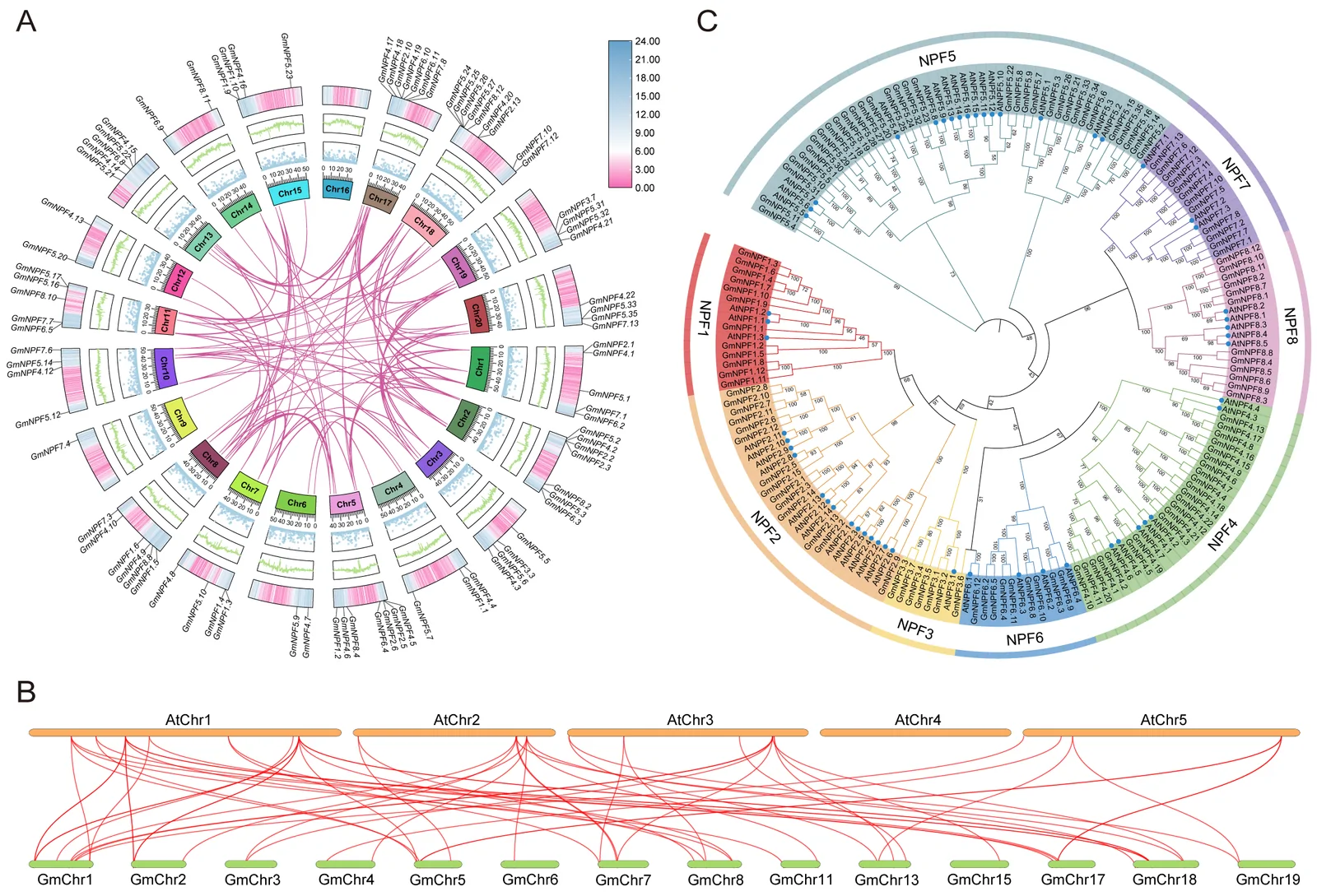
Analysis and illustration of collinearity and Phylogenetics of the *GmNPF* gene family. **(A)** Displays the intra-species collinearity of *GmNPF* genes in soybean (*Glycine max*). The pink lines in the inner circle represent synteny relationships among *GmNPF* genes. From inner to outer, the first layer displays soybean chromosomes, the second layer presents SNPs, the third layer illustrates GC content profiles, the fourth layer illustrates gene density heatmaps, and the outermost layer indicates gene positions. **(B)** The inter-species collinearity between soybean and *Arabidopsis thaliana*, the red lines indicate syntenic *NPF* gene pairs. **(C)** Phylogenetic analysis of *NPF* members from *Glycine max* and *Arabidopsis thaliana*. The phylogenetic tree was constructed using MEGA 11 with the neighbor-joining method based on full-length amino acid sequences (1000 bootstrap replicates). Bootstrap values are shown as percentages on the branches.

To elucidate the evolutionary mechanisms underlying the expansion of the *GmNPF* family. According to the chromosomal locations, the *GmNPF* members were identified on nineteen chromosomes (except for chromosome 16) and distributed unevenly, over 80% of these genes tending to be localized in euchromatic regions with high gene density near the chromosome ends (**Figure 1**). Among these chromosomes, chromosome 18 contained the largest number of *GmNPF* members with 16, whereas chromosomes 6 and 9 each contained only two *GmNPF* genes. This uneven chromosomal distribution might be attributed to multiple rounds of genome duplication events during soybean evolution (Severin et al., 2011; Schmutz et al., 2010). To verify this hypothesis, we systematically analyzed duplication events within the *GmNPF* family. In total, 13 dispersed, 3 proximal, 20 tandem and 85 segmental duplication events were identified, along with one singleton designated *GmNPF5.4* (**Figure 2A**). Furthermore, inter-species synteny analysis between soybean and *A. thaliana* was performed to assess the evolutionary conservation of *NPF* genes across species. A total of 58 soybean *GmNPF*s were found to correspond to 24 *Arabidopsis* orthologs, resulting in multiple *GmNPF* genes mapping to single *Arabidopsis* genes. Most *GmNPF* genes share a clade with their counterpart *AtNPF* genes (**Figure 2B**). This one-to-many homology pattern indicates a close evolutionary relationship between soybean and *Arabidopsis NPF* family members, suggesting that these orthologs may retain core functional characteristics inherited from their common ancestral genes (Liu et al., 2022; Wen et al., 2020).

To further reveal the homology among *GmNPF* members, we aligned soybean and *Arabidopsis* NPF peptide sequences using ClustalW in MEGA with default parameters and constructed a phylogenetic tree using the Neighbor-Joining (NJ) method. Our results revealed that, based on the phylogenetic topology and conserved structural characteristics, *GmNPF* members were grouped into 8 subfamilies, designated *GmNPF1* to *GmNPF8*. Members from the same subfamily were grouped in both *G. max* and *A. thaliana* (**Figure 2C**). Among these subfamilies, *GmNPF5* contains the largest number, with 35 members, whereas *GmNPF3* contains the fewest, with only seven members. Notably, a similar expansion pattern was observed in *Arabidopsis* (Léran et al., 2014), suggesting that the diversification of *NPF* subfamilies occurred before the divergence of the Fabaceae and Brassicaceae lineages. The maintenance of this pattern over approximately 100 million years of independent evolution implies that each subfamily has acquired distinct and evolutionarily conserved functions, thus allowing the potential functions of *GmNPF* genes to be inferred based on their *Arabidopsis* homologs (Wen et al., 2020).

### 3.3 The gene structure and conserved domain of the GmNPF gene family

**Figure 3:**
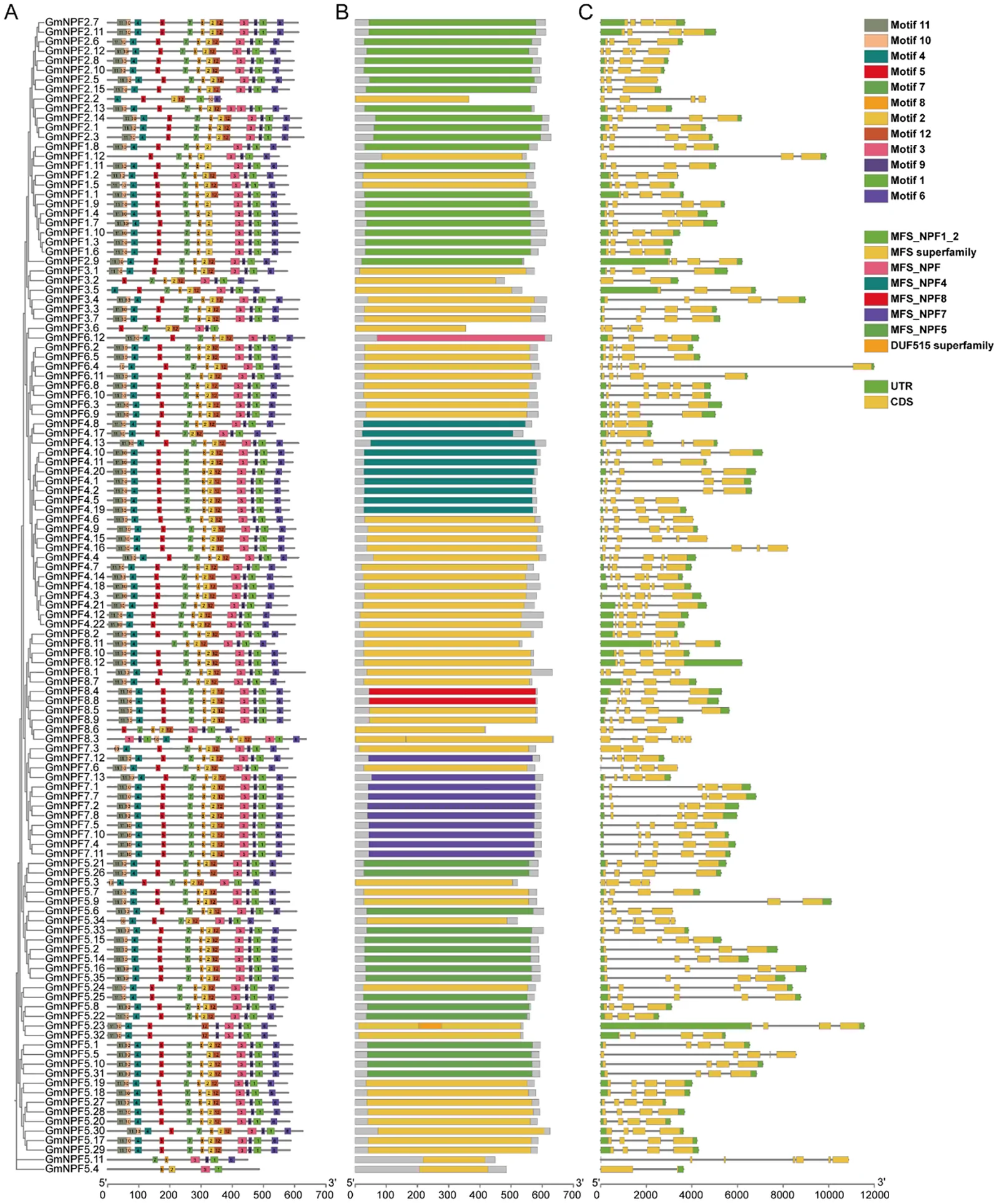
Conserved motif, domain and gene structure of the soybean *GmNPF* gene family. **(A)** Motif analysis was performed using the MEME online tool. **(B)** Conserved domain analysis was conducted with the NCBI Batch CD-search tool. **(C)** In the gene structure schematic, green regions represent untranslated regions (UTRs), yellow regions indicate coding sequences (CDSs) and uncolored lines denote introns.

Conserved domains and motifs are expected to reflect the evolutionary conservation of GmNPF members. A total of 12 conserved motifs were identified, among which motifs 1, 3, 4, 6 and 11 were annotated as belonging to the MFS general substrate transporter-like domains. As shown in **Figure 3**, most GmNPFs harbored a complete set of these motifs. However, some proteins, including GmNPF2.2, GmNPF1.12, GmNPF3.2, GmNPF3.5, GmNPF3.6, GmNPF6.4, GmNPF8.6, GmNPF7.3, GmNPF5.3, GmNPF5.34, GmNPF5.11 and GmNPF5.4, contained only 2–4 of these functional motifs, suggesting that most motif deficient paralogs may have undergone motif loss due to relaxed selection pressure, subfunctionalization or adaptation to new environments (Subramaniam et al., 2013; Lynch and Force, 2000; Cheng et al., 2014). By contrast, GmNPF8.3 possessed six MFS domains; the expansion of MFS domains in GmNPF8.3 represents a rare evolutionary event potentially linked to intragenic domain duplication events and neofunctionalization (Manning and Scheeff, 2010). In addition, the GmNPF subfamily had varied superfamily domain regions, including MFS-NPF1-2, MFS-NPF, MFS-NPF4, MFS-NPF8, MFS-NPF7, MFS-NPF5 and MFS superfamily (**Figure 3**), which is characterized by a typical MFS fold of 12 transmembrane helices (Santin et al., 2021). Besides, a region in GmNPF5.23 belongs to the DUF515 superfamily, suggesting that it may be a chimeric protein. The DUF515 (pfam04415) domain is widely distributed in archaea and is classified as a transmembrane protein, although its function remains largely unknown.

For gene structure analysis, exon numbers differed markedly among *GmNPF* subfamilies. *GmNPF4*, *GmNPF6*, *GmNPF7* and *GmNPF8* possessed more exons, with an average of five, whereas *GmNPF1*, *GmNPF2*, *GmNPF3* and *GmNPF5* had fewer exons, with an average of four. Genes with abundant exons exhibit higher structural complexity and tend to evolve novel biological functions, whereas those with fewer exons maintain relatively conservative structures and perform stable basal functions (Liu et al., 2020a). The *GmNPF5* subfamily was the largest, with exon numbers varying from 1 to 6, reflecting high structural dynamics and frequent intron gain/loss events. Some of the genes contained the long fragment of introns, like *GmNPF1.12*, *GmNPF6.4*, *GmNPF4.16*, *GmNPF5.9*, *GmNPF5.5* and *GmNPF5.11*, which increased transcriptional cost and reduced expression efficiency (Castillo-Davis et al., 2002), but enhanced structural stability and evolutionary conservation (Gorlova et al., 2014). Furthermore, approximately two-thirds of genes had both up and downstream Untranslated Regions (UTRs). This structural diversity in UTRs modulates the accessibility of miRNAs, RNA-binding proteins and translational machinery, and may underlie the differential regulation of transcript stability, translation efficiency and tissue-specific expression among *GmNPF* subfamilies (Mayr, 2016).

### 3.4 Cis-regulatory elements in promoters of GmNPF genes

Upstream cis-regulatory elements (CREs) serve as critical binding sites for transcription factors (TFs) and epigenetic modifications (Wittkopp and Kalay, 2012). Analysis of these regulatory elements is essential for understanding gene expression patterns. To identify CREs for *GmNPFs*, we retrieved the 2-kb upstream regions of *GmNPF* genes in the W82 reference genome and analyzed them with PlantCARE. In total, 34 cis-regulatory elements were identified. In addition to the core elements (CAAT-box and TATA-box), these elements could be specifically categorized into four distinct classes: hormone response elements (GARE-motif, P-box, TATC-box, TGTCA-motif, TGACG-motif, TCA-element, ERE, ABRE, AuxRE, TGA, AuxRR-core), stress response elements (TC-rich repeats, LTR, WUN-motif, ARE, GC-motif, DRE, MBS), transcription regulation (DRE, MBS, MYB binding site, W box, AT-rich element, F-box, MBSI, A-box) and plant development (MSA-like, HD-Zip, CAT-box, circadian, O2-site, Prolamin-box, GCN4-motif, AACA-motif, RY-element, CCAAT-box) (**Supplementary Figure 1**). Some were documented in the regulation of NUE or N deficiency stress, such as MYB, which regulates nitrate uptake by binding to MYB binding sites (Zhang et al., 2021). Notably, promoter regions for all soybean *AAP* genes contained MYB-binding sites, which are involved in regulating gene expression (Li et al., 2019). Functionally, the RY-element and CCAAT-box are two indispensable seed development-related cis-elements. The RY-element functions as a specific binding site for B3-type transcription factors to modulate seed development and maturation (Guerriero et al., 2010), while the CCAAT-box acts as a conserved regulatory motif that cooperates with other seed-specific elements to fine-tune the transcription of genes driving seed growth progression (Jo et al., 2019).

These cis-regulatory elements act as putative targets for various signaling pathways, including phytohormones, stress responses and plant growth and development, to regulate the expression of *GmNPFs*. It has been reported that the ethylene and jasmonic acid signaling pathways regulate the expression of *AtNPF7.3* and *AtNPF7.2* through the hub gene *EIN3*/*EIL1*, thereby promoting NO_3_^-^ accumulation in roots (Zhang et al., 2014; Lin et al., 2008; Chen et al., 2012). In addition, we also observed that four elements, including abscisic acid response elements (ABRE), ethylene response elements (ERE), salicylic acid response elements (TCA-element) and trauma response elements (WUN-motif) were commonly identified in the promoter regions of over half of the *GmNPFs*, and the distribution in the region varied greatly (**Supplementary Figure 1**), indicating that the *GmNPF* family likely play functional roles within these pathways in response to abiotic stressors (Wu et al., 2025).

### 3.5 The GmNPFs display clear tissue expression patterns

**Figure 4:**
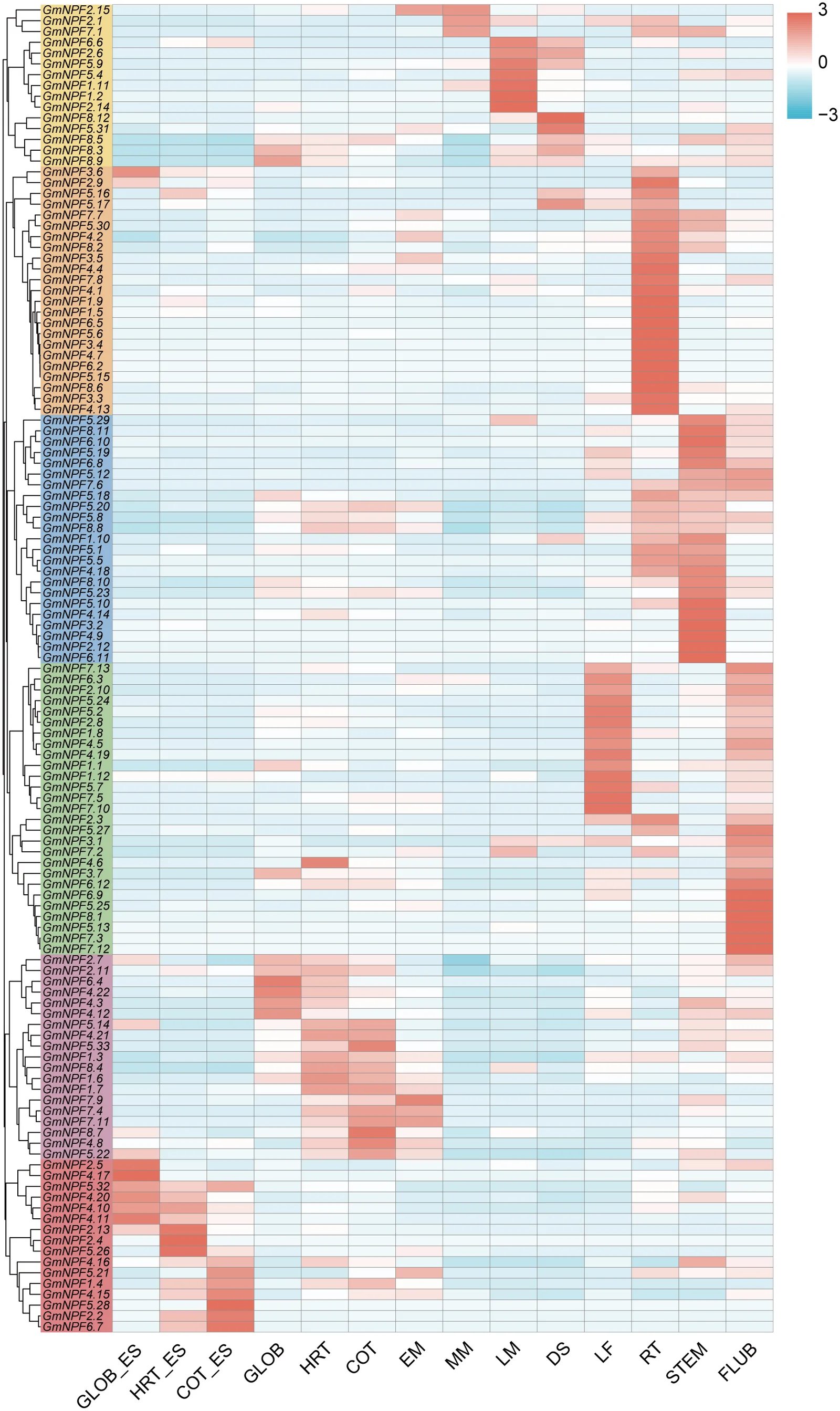
Expression pattern of the *GmNPF* genes in different tissues. The data were obtained from the RNA-Seq data (seedgenenetwork.net). RT-roots; STEM-stems; LF-leaf; FLUB-floral bud; GLOB-globular stage; HRT-heart stage; COT-cotyledon stage; EM-early maturation stage; MM-mid maturation stage; LM-late maturation stage; DS-dry seed; GLOB_ES-globular stage endosperm; HRT_ES-heart stage endosperm; COT_ES-cotyledon stage endosperm.

To gain insight into the expression profiles of *GmNPF* genes, we analyzed their transcript abundances across diverse soybean tissues [RT (roots), STEM (stems), LF (leaf), FLUB (floral bud)] and developing seeds at sequential developmental stages [GLOB (globular stage), HRT (heart stage), COT (cotyledon stage), EM (early maturation stage), MM (mid maturation stage), LM (late maturation stage), DS (dry seed), GLOB_ES (globular stage endosperm), HRT_ES (heart stage endosperm), COT_ES (cotyledon stage endosperm)] via reanalysis of publicly available RNA-Seq datasets retrieved from seedgenenetwork.net (**Figure 4**). Distinct spatiotemporal expression patterns were evident for most *GmNPF* genes across the tested tissues and seed developmental stages, whereas three members (*GmNPF6.1*, *GmNPF5.3*, *GmNPF5.11*) exhibited no detectable transcripts in any of the assayed samples. On the basis of hierarchical clustering of expression patterns, the identified *GmNPF* genes were categorized into six discrete groups. Genes belonging to Groups 1 and 5 were predominantly expressed during seed maturation stages and in dry seeds, implying their potential roles in the translocation of nitrogen-containing compounds in soybean seeds. Group 2 comprised 23 genes with preferential expression in roots, suggesting their involvement in root-mediated nitrogen uptake from the rhizosphere. Group 3 genes were ubiquitously expressed in roots, stems and floral buds; notably, *GmNPF4.14*, *GmNPF3.2*, *GmNPF4.9*, *GmNPF2.12* and *GmNPF6.11* displayed the highest transcript levels in stems relative to other assayed tissues, suggesting that these genes may be involved in long-distance transport. Group 4 contained 28 genes that were highly expressed in leaves and floral buds, indicating possible tissue-specific roles in nitrogen metabolism or allocation within these organs. Group 6 genes were mainly expressed in the endosperm. The endosperm, though ephemeral in soybean, functions as a crucial hub for the uptake, temporary accumulation and subsequent remobilization of nutrients toward the developing cotyledons (**Figure 4**). The enrichment of *GmNPF* transcripts in this tissue implies that these genes may facilitate the flux of nitrogenous compounds across the endosperm, thereby contributing to the nitrogen supply for seed protein accumulation (David et al., 2014). Collectively, these expression profiles provide important clues for prioritizing candidate *GmNPF* genes for further expression validation and functional characterization, laying a foundation for dissecting their precise biological roles in soybean nitrogen use efficiency.

### 3.6 Expression patterns of GmNPF genes under low nitrogen

**Figure 5:**
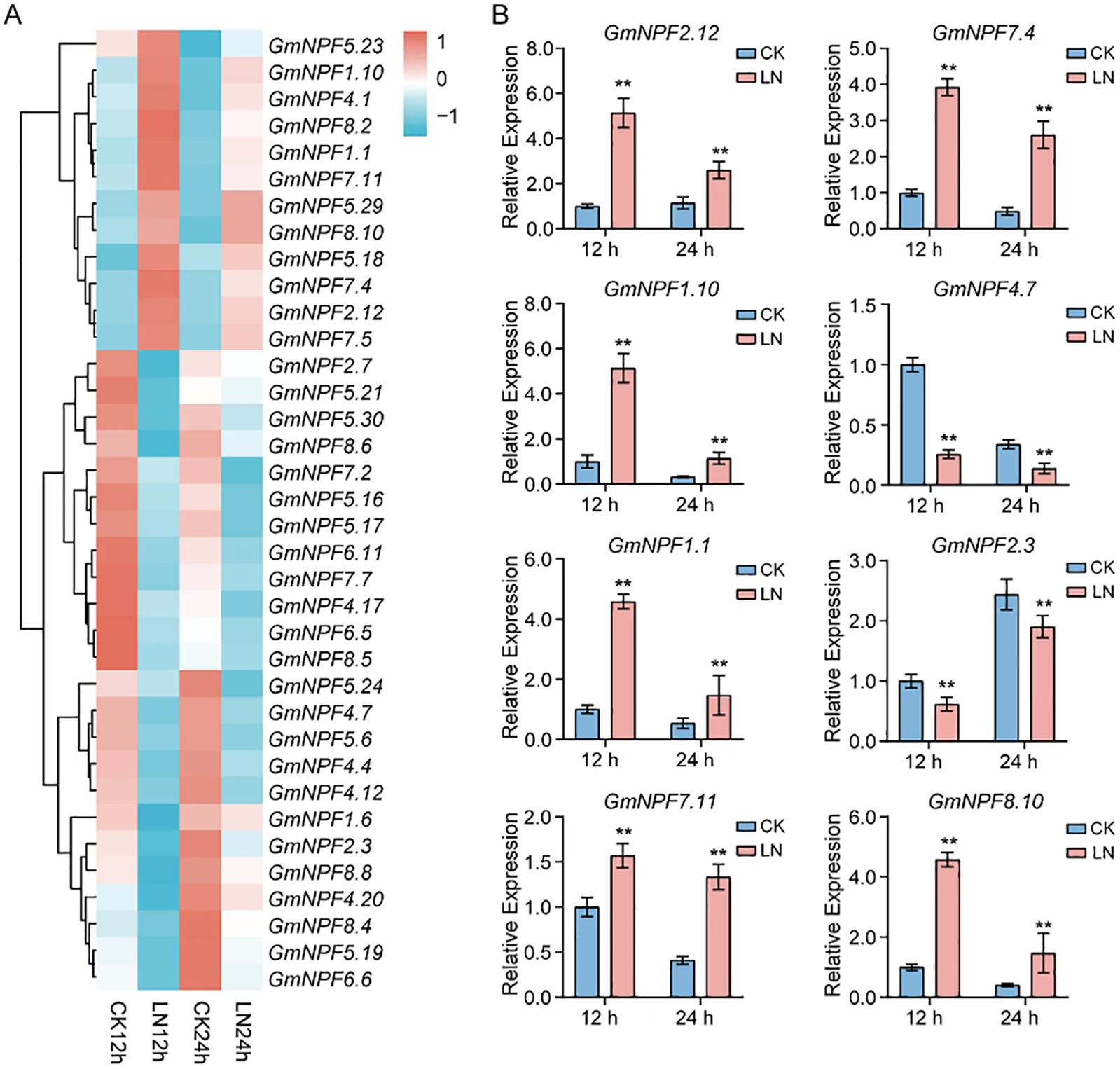
The expression levels of the *GmNPF* genes in soybean roots under CK (NN) and LN conditions at 12h and 24h after treatment. **(A)**. The expression data were obtained from RNA-seq data and shown as log_2_^FPKM^ values. **(B)**. The selected genes were expressed by qRT-PCR assay. Soybean *TUB4* was used as an internal control. Data are the mean ± SE of three biological replicates. Asterisks indicate statistically significant differences based on Student’s t-test (*a =0.05, **a = 0.01).

In the early growth stage of soybean, roots serve as the primary organ for nitrogen absorption, and the expression of GmNPF genes in roots is critical for nitrogen uptake and transport. To elucidate the responses of GmNPFs to low nitrogen (LN) stress, transcriptome profiling was performed on root tissues exposed to hydroponic culture with normal nitrogen (7.5 mM) or LN (0.75 mM) conditions at 12 h and 24 h. Notably, 36 genes exhibited significant differential expression compared to the control under nitrate deprivation at both time points (**Figure 5A**). Among these, 12 genes showed significantly increased expression, while the remaining 24 genes were downregulated. Half of these genes showed the highest expression levels in root tissues (**Figure 4**). To validate the above transcriptomic results, we quantified the expression profiles of 11 representative GmNPF genes via reverse transcription quantitative PCR (RT-qPCR), all of which exhibited differential expression. As shown in **Figure 5B**, the transcript levels of GmNPF2.12, GmNPF7.4, GmNPF7.11, GmNPF1.10, GmNPF3.5, GmNPF4.14, GmNPF8.10 and GmNPF1.1 were consistent with the transcriptomic analysis, showing sustained upregulation in roots under low nitrogen (LN) stress at both 12 h and 24 h, whereas GmNPF4.7, GmNPF5.32 and GmNPF2.3 were markedly downregulated under the same treatment conditions. Homologous NPF members have been reported to mediate ion transport under nitrogen deficiency. For instance, AtNPF7.3 functions in potassium transport in Arabidopsis roots upon low nitrogen exposure (Drechsler et al., 2015). Collectively, these results confirm that these GmNPF genes are responsive to nitrogen availability, providing a foundation for systematic dissection of GmNPF family expression dynamics under LN conditions.

### 3.7 Differentially expressed GmNPF genes influenced by two major high protein QTL genes

Given that *NPF* genes were involved in NUE and nitrogen transport and the role of increasing protein content (Fang et al., 2017; Meng et al., 2024), we ask if any of the *GmNPF* genes were involved in the regulation of seed protein content. A straightforward way is to examine whether any of the *GmNPFs* were regulated by protein controlling genes *POWR1* and *SWEET39* (Goettel et al., 2022; Zhang et al., 2020; Sun et al., 2025; Wang et al., 2020b). With the availability of soybean transgenic plants for *POWR1* overexpression (OE) and a double mutant of *sweet10a/b (sweet39)* that both exhibited increases in protein content, we evaluated the expression levels in them in comparison with the wild plants.

**Figure 6:**
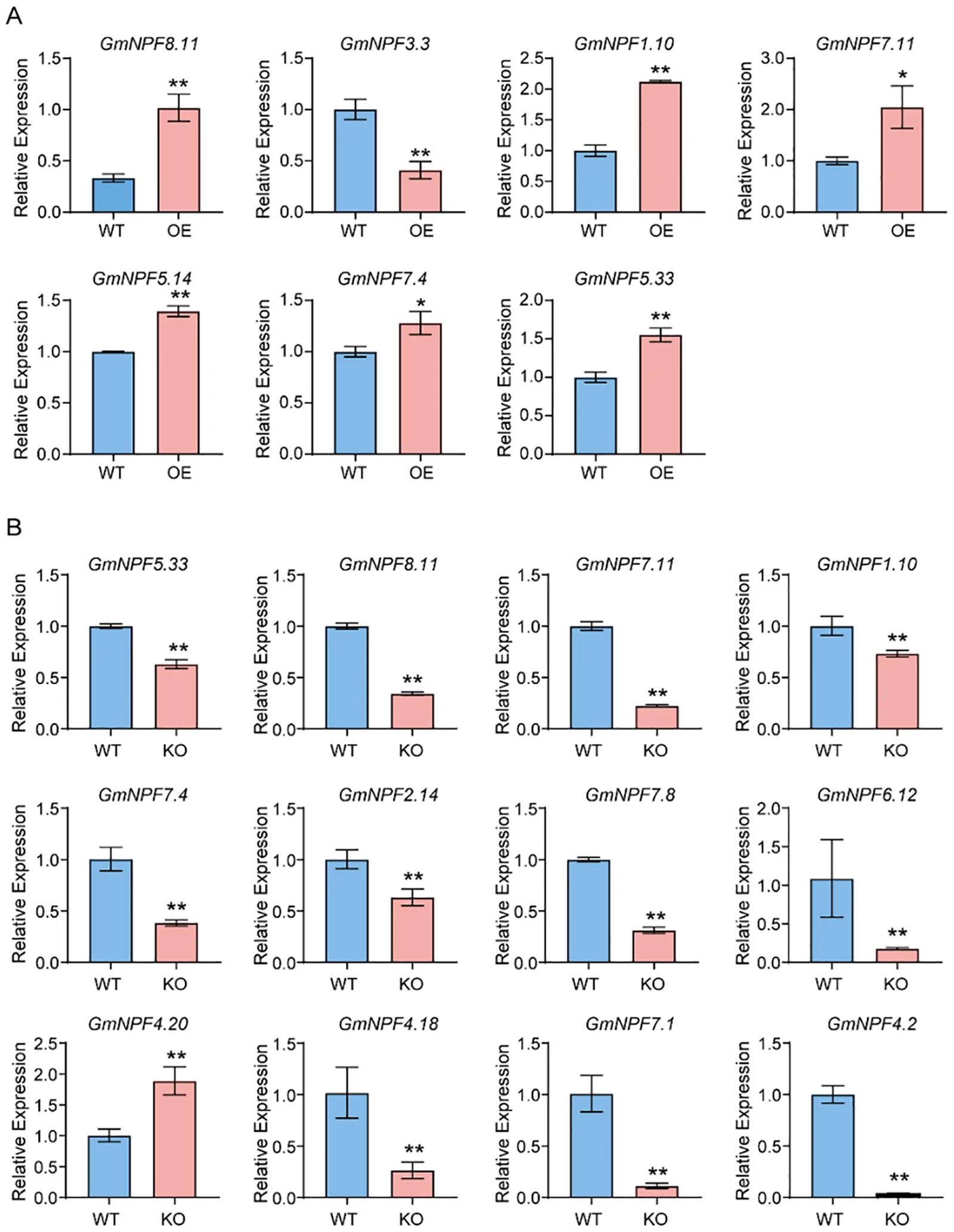
Expression analysis of *GmNPF* family genes in *POWR1*-OE and *SWEET39*-KO soybean plants. **(A)**. Relative expression levels of selected *GmNPF* genes in WT and *POWR1*-OE soybean plants. **(B)**. **(B)** Relative expression levels of selected *GmNPF* genes in WT and *SWEET39*-KO soybean plants. The selected genes were expressed by the qRT-PCR assay. Soybean *TUB4* was used as an internal control. Data are the mean ± SE of three biological replicates. Asterisks indicate statistically significant differences based on Student’s t-test (*a =0.05, **a = 0.01).

To comprehensively investigate the effect of *POWR1*-OE on *GmNPF* expression, we performed transcriptome sequencing (RNA-seq) on seed coats at 30 days after flowering (DAF), a critical stage for amino acids transport from maternal tissues to growing embryo (Rainbird et al., 1984). Based on the consistent expression trends between two biological replicates, a total of 38 differentially expressed *GmNPF* genes were identified in wild type (WT) and *POWR1*-OE plants (**Supplementary Figure 2A**). Most of these genes exhibited high expression levels in stems and seeds (**Figure 4**). Among these, genes of the *GmNPF5* family were abundantly detected, and given that *AtNPF5.5* has been reported to function in nitrogen accumulation within embryos in *Arabidopsis* (Léran et al., 2015), RT-PCR analysis of soybean *GmNPF5* family genes revealed significantly elevated transcript levels of *GmNPF5.14* and *GmNPF5.33* in OE lines (**Figure 6A**). Furthermore, considering that the maize *NPF7.9* homolog participates in nitrate allocation from vegetative organs to seeds (Wei et al., 2021). RT-PCR assays detected upregulation of *GmNPF7.4* and *GmNPF7.11* as well as downregulation of *GmNPF7.9* in the mutant (**Figure 6A**), consistent with the transcriptomic analysis (**Supplementary Figure 2A**).

To further elucidate the regulatory link between *GmSWEET39* and *GmNPF* genes, *GmSWEET39*-KO mutants were generated in the W82 background, followed by RNA-seq analysis. A total of 64 differentially expressed *GmNPF* genes were identified (**Supplementary Figure 2B**), among which 29 were also differentially expressed in *POWR1-OE* lines (**Supplementary Figure 2C**). RT-PCR verification of these overlapping genes revealed significantly decreased expression levels of *GmNPF5.33*, *GmNPF8.11*, *GmNPF7.11*, *GmNPF7.4* and *GmNPF1.10* (**Figure 6B**). Previous studies have shown that mutation of *OsNPF4.1* leads to defective panicle development, smaller seeds and reduced grain number in rice (Li et al., 2009). Accordingly, differentially expressed genes belonging to the *GmNPF4* family were examined via RT-PCR, which showed significantly reduced transcript levels of *GmNPF4.18* and *GmNPF4.2*, but significantly increased expression of *GmNPF4.20* (**Figure 6B**). Meanwhile, several other significantly regulated genes were detected, among which *GmNPF2.14*, *GmNPF4.18*, *GmNPF7.1*, *GmNPF7.8* and *GmNPF6.12* exhibited decreased transcript abundances in the KO mutants (**Figure 6B**).

**Figure 7:**
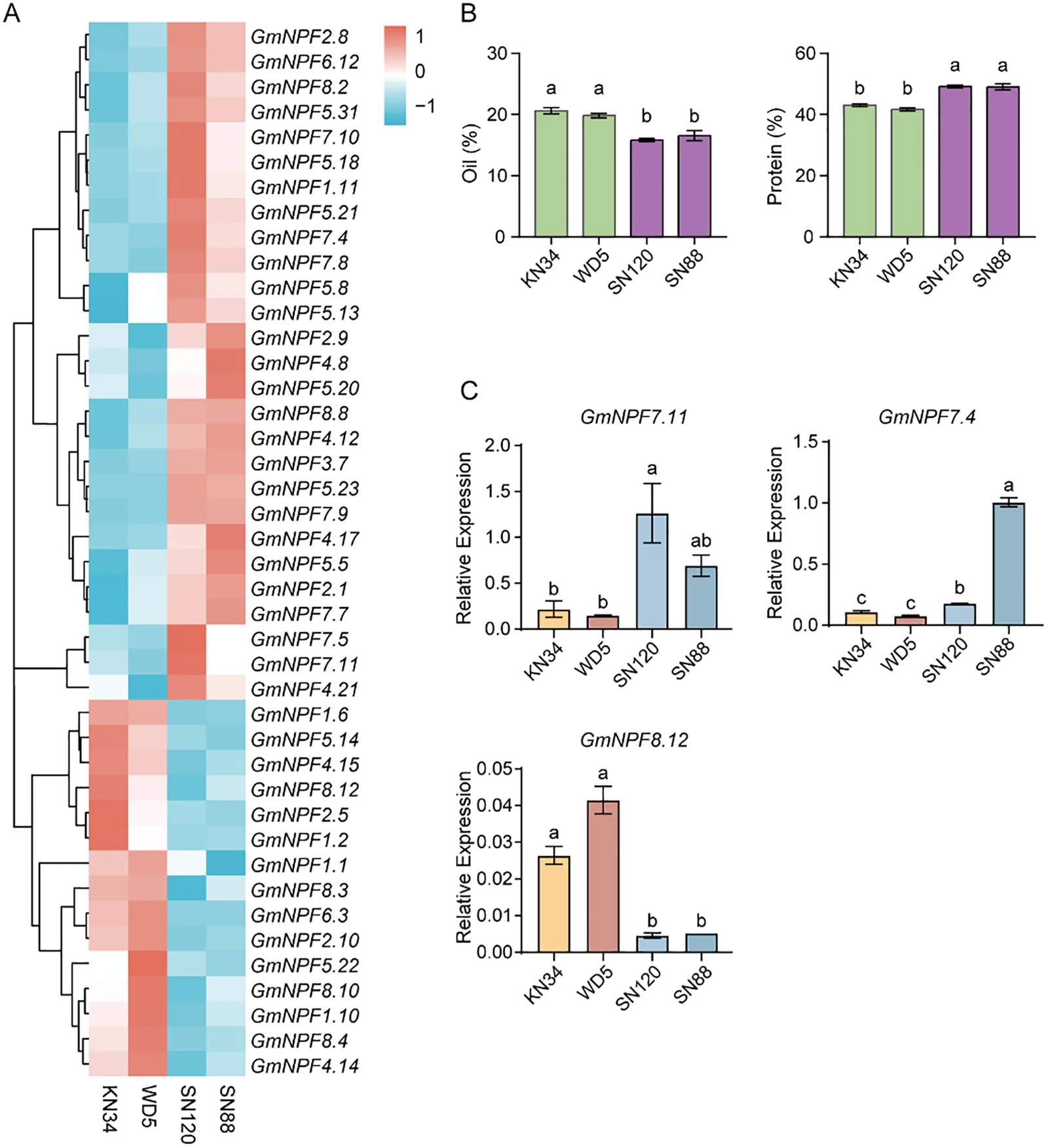
The expression levels of the *GmNPF* genes in soybean seed coat in Kennong34 (KN34), Wodou5hao (WD5), Suinong120 (SN120) and Suinong88 (SN88). **(A)**. The expression data were obtained from RNA-seq data and shown as log_2_^FPKM^ values. **(B)** Seed oil and protein contents of KN34, WD5, SN120 and SN88. **(C)** Relative expression levels of *GmNPF7.11, GmNPF7.4*, and *GmNPF8.12* in four soybean materials. Different lowercase letters (a, b, c, d) above the bars indicate significant differences at *P* < 0.05 according to Tukey's multiple comparison test. Error bars represent standard deviation.

Additionally, to explore whether *GmNPF* genes are associated with variation in seed protein content in soybean cultivars, we selected four soybean cultivars with contrasting protein contents, in which Kennong34 (KN34) and Wodou5hao (WD5) are low protein varieties with 42.33% protein content; Suinong120 (SN120) and Suinong88 (SN88) are high protein varieties with 49.10% protein content (**Figure 7B**). RNA-seq was performed on seed coats at 30 DAF to compare *GmNPF* expression profiles between the two groups, and a total of 42 differentially expressed genes were identified (**Figure 7A**). RT-PCR validation further confirmed that *GmNPF7.11* and *GmNPF7.4* were significantly upregulated in high protein cultivars, whereas *GmNPF8.12* exhibited markedly lower transcript levels (**Figure 7C**), consistent with the transcriptomic analysis.

### 3.8 Haplotype characterization of GmNPF genes for soybean seed traits

**Figure 8:**
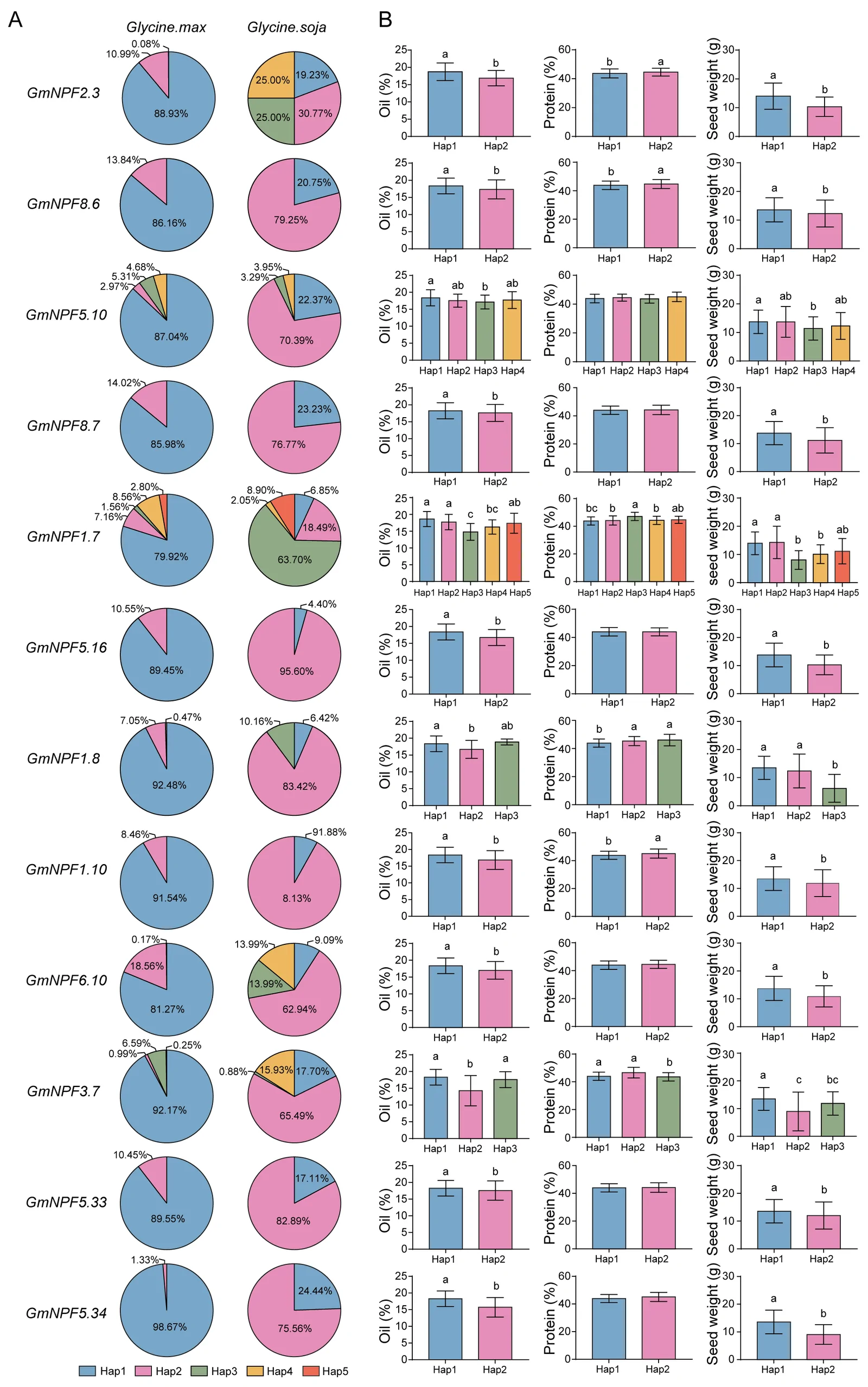
Expression patterns and haplotype effects of soybean *GmNPF* family genes. **(A)** Haplotype frequency distribution of selected *GmNPF* genes in *Glycine max* and *Glycine soja*. **(B)** Associations of major haplotypes of *GmNPF* genes with three key seed traits in *Glycine max*. Different lowercase letters (a, b, c, d) above the bars indicate statistically significant differences among haplotypes at *P* < 0.05, as determined by one-way analysis of variance (ANOVA) followed by Tukey’s multiple range test.

To further explore the potential contribution of variations in members of the *GmNPF* gene family within natural populations to soybean quality, we conducted haplotype analysis of the *GmNPF* gene family. There are significant differences in seed protein levels between cultivated and wild soybeans (Wang et al., 2020b); thus, we analyzed haplotype variations in genes with more than 75% allelic divergence between wild and cultivated soybeans. The results showed that 12 *GmNPF* genes harbor single-nucleotide polymorphisms (SNPs) in the coding sequences (CDS) that show great divergence in allelic frequencies, including *GmNPF2.3*, *GmNPF8.6*, *GmNPF5.10*, *GmNPF8.7*, *GmNPF1.7*, *GmNPF5.16*, *GmNPF1.8*, *GmNPF1.10*, *GmNPF6.10*, *GmNPF3.7*, *GmNPF5.33* and *GmNPF5.34* (**Figure 8**). Based on the genotyping of key genetic variations, Hap1 was identified as the predominant haplotype in *G. max*, with frequencies ranging from 98.67% to 79.92%, whereas Hap2 was primarily the major haplotype in *G. soja*. Notably, the major alleles detected in *G. soja* were largely preserved in *G. max*. Comparative analysis of seed protein content among different haplotypes in cultivated soybean uncovered significant differences in protein levels associated with distinct haplotypes, including *GmNPF2.3*, *GmNPF8.6*, *GmNPF1.7*, *GmNPF1.8*, *GmNPF1.10* and *GmNPF3.7* (**Figure 8B**). For most of these genes, accessions carrying Hap2 contain significantly higher protein content than those carrying other haplotypes, with increases ranging from 2.05% to 5.91%. In contrast, Hap3 of *GmNPF1.7* showed the highest protein content, with an average of 47.1%. We also examined oil accumulation and seed weight across different haplotypes. Hap1 of *GmNPF2.3* displayed the maximum oil content and yield, which were 18.7% and 14 Mg·ha⁻¹, respectively (**Figure 8B**). Consistent with the known trade-off between protein and oil in soybean seeds, higher protein content was accompanied by reduced oil content and seed weight (Schwender et al., 2003). Furthermore, literature mining indicated that all these genes were located within major QTL intervals associated with seed protein, oil and weight in soybean. For example, Asekova et al. (2016) constructed recombinant inbred lines (RILs) and mapped a QTL associated with crude protein content on chromosome 14 named *Crude protein R6 1-3*. Notably, *GmNPF1.8* is located within this mapped interval. Collectively, these findings provide a resource of candidate genes to facilitate the genetic improvement of soybean protein levels via molecular marker-assisted breeding.

## 4 Discussion

Nitrogen is an essential constituent for the synthesis of organic matter in organisms. Higher plants can directly absorb and assimilate exogenous nitrogen, mainly in the form of NO_3_⁻, for growth and metabolism. The *NPF* gene family extensively mediates NO_3_⁻ uptake, regulation, translocation and allocation/remobilization (Léran et al., 2014). Systematic investigation of *NPF* genes promises to enhance nitrogen use efficiency, optimize plant nutritional quality, and advance eco-sustainable agricultural practices. Cultivated soybean serves as a vital source of plant protein for humans, and characterization of its NPF gene family is crucial for improving nitrogen use efficiency and protein synthesis. Previous studies have provided valuable insights into the genome-wide identification and basic characterization of the *GmNPF* gene family in soybean, including both *Glycine max* and *Glycine soja* (Wang et al., 2026; You et al., 2020). However, the present study extends beyond these descriptive analyses by demonstrating that *GmNPF* genes are directly regulated by *POWR1* and *SWEET39*, two major high-protein QTLs, and that allelic variations in six *GmNPF* genes are associated with seed protein content variation in cultivated soybean. These findings place *GmNPF* genes within the regulatory network of seed quality traits, providing new insights into the molecular mechanisms underlying nitrogen transport during seed development.

A total of 126 NPF proteins in soybean were identified, which is more than the *G. soja* (120 *NPFs*) (You et al., 2020) and implies a genome expansion. The preferential localization of *GmNPF* genes in distal euchromatic regions is consistent with the notion that chromosomal distal regions feature high recombination frequency and active DNA double-strand break repair, both of which facilitate the occurrence of gene duplication events (Allitto et al., 1990; Boyd, 1990). In support of this, our duplication event analysis revealed that segmental duplications were the predominant type and played a pivotal role in driving the expansion of the *GmNPF* gene family, a pattern also reported for *NPF* families in other plant species (Wen et al., 2020). Notably, *GmNPF5.4* with no detectable homologs in the soybean genome, suggesting that it may represent a soybean-specific gene that arose after the divergence of soybean from other legume lineages (Ambrosino et al., 2016). Since the functions of *NPF*-related genes have been extensively studied in the model plant species *Arabidopsis thaliana*, we constructed a phylogenetic tree of *NPF* genes from soybean and *Arabidopsis* and performed collinearity analysis. The results revealed high levels of homology between the two species (**Figure 2B**), providing valuable insights for predicting the functions of soybean *NPF* genes based on their *Arabidopsis* counterparts.

Analysis of gene promoters holds significant theoretical and practical value, it is possible to decipher the regulatory networks governing gene expression in specific tissues, developmental stages, or under environmental stress. Moreover, promoter analysis provides critical targets for molecular design breeding, offering a theoretical foundation for crop genetic improvement and biotechnological innovation (Villao-Uzho et al., 2023). For example, *ethylene response factors* (*ERF*) bind to the GCC boxes in the *AtNRT1.8 (AtNPF7.2)* promoter region and upregulate *AtNRT1.8* and downregulate *AtNRT1.5* (*AtNPF7.3*), which collectively modulate stress tolerance and plant growth (Zhang et al., 2014). MYB59 can bind to the promoter of *AtNPF7.3* and is involved in K^+^ and NO_3_^-^ transport under low K^+^ and low NO_3_^-^ conditions (Du et al., 2019). This study identified MYB transcription factor binding sites in the promoters of several *GmNPF* genes, suggesting that these genes may also participate in nitrate transport under low-nitrogen conditions (**Supplementary Figure 1**). In addition, RY-element and CCAAT-box cis-acting elements associated with soybean seed development were also identified in the promoters of *GmNPF* genes (**Supplementary Figure 1**), and most of the genes were highly expressed in the seed (**Figure 4**), suggesting that *GmNPF* genes may be involved in soybean seed development. Previous studies have reported that the *ARF10–STF3* regulatory cascade directly binds to the G-box (CACGTG) motif in the *GmNPF2.13* promoter and activates *GmNPF2.13* in soybean, thereby strengthening nitrate uptake and further regulating the accumulation of nutrients in soybean seeds (Xu et al., 2026), Consistently, our tissue expression profiling showed that *GmNPF2.13* displayed the maximum transcript abundance in HRT_ES, in agreement with earlier reports (**Figure 4**).

Genes of the *NPF* family mediate the absorption, transport and subsequent utilization of NO_3_^-^, and are thus widely expressed in roots, stems, leaves, flowers and seeds. The biological functions of the *NPF* family have been well characterized in *Arabidopsis thaliana*. For example, *AtNPF7.2* facilitates xylem loading of nitrate, and it is specifically expressed in root pericycle cells adjacent to xylem vessels (Lin et al., 2008), while *AtNPF7.3* and *AtNPF2.9*, expressed in root xylem parenchyma and phloem companion cells, respectively, coordinate nitrate unloading from xylem (Wang and Tsay, 2011; Lin et al., 2008). Collinearity analysis and phylogenetic tree construction revealed that *AtNPF7.2* and *AtNPF7.3* share the highest homology with *GmNPF7.8* (**Figure 2B**, **Figure 2C**). Transcript abundance analysis showed that *GmNPF7.8* displayed the highest expression level in roots (**Figure 4**), implying that it may possess conserved biological functions with its *Arabidopsis* homologs. Notably, *GmNPF6.5* clusters within the same phylogenetic clade and shares significant collinearity with *AtNPF6.3*, a well-characterized *Arabidopsis* homolog known to regulate multiple physiological processes, including root to shoot NO_3_⁻ translocation, lateral root development, nascent organ growth, flowering time modulation and stress responses. Meanwhile, *GmNPF6.5* exhibited the highest expression abundance in seeds (**Figure 4**), suggesting that it may also participate in the above regulatory processes (Nedelyaeva et al., 2024). *AtNPF1.2*, *AtNPF1.1* and *AtNPF2.13* are predominantly expressed in phloem companion cells of leaf veins, contributing to efficient nitrate remobilization and redistribution (Hsu and Tsay, 2013; Fan et al., 2009). *AtNPF1.1* and *AtNPF1.2* show the closest phylogenetic relationship with *GmNPF1.1* and *GmNPF1.1* also had the highest expression level in leaves (**Figure 4**). These results indicate that *GmNPF1.1* is likely involved in nitrate transport in soybean leaves. *AtNPF2.12* exhibits exclusive expression in the vascular bundles of reproductive organs, including siliques and fruit stalks, where it regulates nitrate-mediated early embryo development in *Arabidopsis* (Almagro et al., 2008). *AtNRT2.7* is highly expressed in seeds and is involved in nitrate accumulation (Chopin et al., 2007). In addition, *AtNPF2.12* shares the highest homology with soybean *GmNPF2.1* and *GmNPF2.3* (**Figure 2B**). Among them, *GmNPF2.1* showed dominant expression in seeds (**Figure 4**), which implies its potential involvement in nitrogen accumulation during soybean seed development.

Under nitrogen-deficient conditions, plants optimize nitrogen acquisition and allocation by regulating the expression of nitrogen transporter genes to adapt to nitrogen limitation. While previous studies have primarily focused on the response mechanisms of high-affinity nitrate transporter gene families (e.g., *NRT2*) under low N availability (Takatoshi and Krapp, 2016), the molecular mechanisms through which other nitrogen transporter families (particularly *NPF*) participate in nitrogen redistribution under nitrogen-deficient conditions remain underexplored. In this study, we analyzed the expression patterns of the *GmNPF* gene family under nitrogen-deficient conditions using transcriptomics and qRT-PCR. The results revealed significant upregulation of *GmNPF7.4*, *GmNPF7.11* and *GmNPF1.10* at both 12 and 24 hours after nitrogen treatment (**Figure 5A**). Previous research has demonstrated that *AtNRT1.5*/*NPF7.3* is involved in the distribution of nitrate within the plant (Almagro et al., 2008). In wheat, the expression of *TaNPF7.1* was significantly reduced after 3 and 6 days of N-starvation (Buchner and Hawkesford, 2014), which contrasts with our findings. This suggests that functional divergence may have occurred in the *NPF7* subfamily, leading to the evolution of distinct nitrogen response strategies across species. In *Arabidopsis*, the *NPF1* gene mediates the translocation of root-derived NO_3_⁻ to mature leaf phloem for subsequent allocation to young leaves (Hsu and Tsay, 2013). Single-cell transcriptome analyses further reveal that *AtNPF1* exhibits the highest expression in procambium cells, suggesting its involvement in NO_3_⁻ loading within both developing root meristems and mature phloem/xylem vasculature (Lhamo and Sheng, 2021). Building on these findings, we hypothesize that *GmNPF1.10* in soybean may functionally parallel this process during low nitrogen adaptation.

The soybean seed coat is the central hub for the transport of embryonic substances in soybeans. It is not only a physical barrier between the maternal plant and the embryo, but also a key node for the targeted allocation of essential nutrients such as carbon and nitrogen sources to the endosperm, connecting the overall nutrient metabolism of the plant with seed development (Moïse et al., 2005). Numerous studies have shown that *NPF* genes are involved in nitrogen accumulation in the embryo. *AtNPF5.5*, *AtNPF2.12* and *AtNRT2.7* are involved in nitrate transport in soybean seeds (Almagro et al., 2008; David et al., 2014; Léran et al., 2015); *ZmNPF7.9* and *ZmNPF8.8* are involved in NO^3-^ accumulation and transport peptide in seeds, respectively (Wei et al., 2021; Tnani et al., 2013). However, research on the role of *NPF* genes in nitrogen transport in soybean seeds remains scarce. *POWR1* and *GmSWEET39* are specifically expressed in plant seed coats but adopt distinct nitrogen and carbon allocation patterns (Li et al., 2025; Goettel et al., 2022; Liu et al., 2026; Zhang et al., 2020). Among the common significant difference genes detected by RT-PCR in *POWR1-OE* and *SWEET-KO*, all exhibited opposite expression patterns, indicating that *GmNPF* genes possess distinct expression patterns in response to varying protein contents in soybean seeds (**Figure 6A**, **Figure 6B**). It is speculated that the knockout of Gm*SWEET39* homologous genes affects sugar accumulation in embryos, leading to insufficient carbon supply, reduced oil content and relatively increased protein content (Wang et al., 2020b). Consistent with this, both high protein and low protein groups harbored functional *GmSWEET* genes, thereby exhibiting expression patterns identical to those of the *POWR1*-OE (**Figure 6**, **Figure 7C**).

Natural genetic variation is a fundamental source of crop domestication and genetic improvement. Functional differences among distinct alleles or haplotypes give rise to diverse agronomic traits, and such haplotype effects possess crucial application value in modern breeding. For example, a short sequence insertion in the promoter region of *PC08*, a key gene regulating soybean protein content, elevates its transcriptional level and promotes seed storage protein accumulation by modulating ABA content. Introgression of the high protein allele *PC08^Ins^* into the cultivated soybean variety Heinong 35 significantly increased seed protein content (Liu et al., 2025). Thus, natural variation in functional genes can profoundly influence soybean protein content. The accumulation of soybean protein relies on the processes of nitrogen uptake, transport and remobilization, in which the *GmNPF* family genes encode proteins involved in key steps of nitrogen transport. Consequently, natural variation within this family may also affect the final protein content of soybean, either synergistically with or independently of the mechanisms described above. We performed haplotype and phenotypic analyses on *GmNPF* genes with allele > 75% in cultivated and wild soybeans that exhibit extremely large differences in seed protein and oil (Patil et al., 2018). The results showed that there were significant differences in oil content among different haplotypes of the screened *GmNPF* genes (**Figure 8**).

Combined with the analyses in this study, we also found that the expression level of *GmNPF1.10* was significantly different under low nitrogen utilization conditions, as well as in soybean mutants of protein-related genes and in varieties with different protein contents. Furthermore, this gene carries genetic polymorphisms that contribute to obvious phenotypic divergence. Therefore, this study can provide important genetic resources and theoretical support for the synergistic improvement of high yield, superior quality, and high nitrogen use efficiency in soybean, and lay a foundation for the subsequent breeding of new soybean varieties with high nitrogen efficiency and high quality through marker-assisted selection or gene editing.

However, a limitation of this study is that we only applied a single tissue in the investigation of low N responsiveness, it is worth investigating the expression of *GmNPF* genes across all relevant tissues, which could lead to a more comprehensive understanding of the *GmNPFs* expression and overlook potential tissue-specific regulatory mechanisms to low N stress. From the perspective of research needs for regulating soybean seed protein content, this study only focuses on the correlation between gene expression characteristics and phenotypes. The interaction between *GmNPF* genes and regulatory factors such as *POWR1*, as well as the molecular mechanism of downstream regulatory pathways, has not been thoroughly verified. The application of the research conclusions needs to be further supported by more genetic transformation experiments.

## 5 Conclusions

This study systematically deciphered the evolutionary characteristics and expression regulation patterns of the soybean *NPF* gene family. Through collinearity analysis, we identified gene duplication events, while phylogenetic tree construction classified them into eight conserved subfamilies. Domain architecture analysis confirmed that all members contain the characteristic NRT1/PTR family motifs. Promoter analysis revealed abundant hormone and stress-responsive cis-elements, with particular enrichment of ethylene/ABA-related elements. Expression profiling demonstrated that some genes (e.g., *GmNPF7.4*/*7.11*/*1.10*) exhibit sustained upregulation under nitrogen deficiency. The analysis of gene expression levels and soybean seed protein content showed that the *GmNPF7.4* and *GmNPF7.11* were significantly positively correlated with seed protein content. Haplotype analysis revealed six genes carrying protein content related haplotypes, namely *GmNPF2.3*, *GmNPF8.6*, *GmNPF1.7*, *GmNPF1.8* and *GmNPF3.7*. These findings not only clarify the evolutionary relationships within the soybean *NPF* gene family but also reveal their potential functional diversification in nitrogen absorption and translocation, thereby providing crucial theoretical foundations for genetic improvement of nitrogen use efficiency in crops.

## Data Availability

The original contributions presented in the study are included in the article/**Supplementary Material**. Further inquiries can be directed to the corresponding author.

## Supplementary material

Supplementary Material List of primers used in this study.

Supplementary Table 1 Soybean GmNPF family and physicochemical properties.

Supplementary Figure 1 Predicted cis-regulatory element in the 2000-bp region upstream of the start codon (ATG) of each *GmNPF* gene. **(A)** Number of cis-acting elements within the 2000 bp upstream region of each gene. **(B)** Distribution of cis-acting elements across the 2000 bp upstream region of each gene.

Supplementary Figure 2 Expression profiles of *GmNPF* genes under *POWR1-OE* and *GmSWEET39*-KO conditions. **(A)** Heatmap showing the expression changes of *GmNPF* genes in wild-type and *POWR1*-OE soybean plants. Color scale represents normalized gene expression values (z-score). **(B)** Expression patterns of *GmNPF* genes in wild-type and GmSWEET39-KO plants. The color gradient corresponds to z-score normalized transcript abundance. **(C)** Venn diagram illustrating the number of differentially expressed *GmNPF* genes responding to *POWR1*-OE, *GmSWEET39*‑KO, and the overlap of co-differentially expressed genes.
